# Radiation resistance due to high expression of miR-21 and G2/M checkpoint arrest in breast cancer cells

**DOI:** 10.1186/1748-717X-7-206

**Published:** 2012-12-05

**Authors:** Nataša Anastasov, Ines Höfig, Iria Gonzalez Vasconcellos, Kristina Rappl, Herbert Braselmann, Natalie Ludyga, Gert Auer, Michaela Aubele, Michael J Atkinson

**Affiliations:** 1 Institute of Radiation Biology, Helmholtz Zentrum Muenchen, German Research Center for Environmental Health, Muenchen, Germany; 2 Institute of Pathology, Helmholtz Zentrum Muenchen, German Research Center for Environmental Health, Muenchen, Germany; 3 Research Unit of Radiation Cytogenetics, Helmholtz Zentrum Muenchen, German Research Center for Environmental Health, Muenchen, Germany; 4 Department of Oncology and Pathology, Karolinska Institute and Hospital, Stockholm, Sweden; 5 Radiation Biology, Institute of Radiation Oncology, Technical University Munich, Munich, Germany

**Keywords:** MiR-21, Breast cancer, Radiation resistance, G2/M checkpoint arrest

## Abstract

**Background:**

There is evidence that the extent of the G2/M arrest following irradiation is correlated with tumour cell survival and hence therapeutic success. We studied the regulation of cellular response to radiation treatment by miR-21-mediated modulation of cell cycle progression in breast cancer cells and analysed miR-21 expression in breast cancer tissue samples with long-term follow up.

**Methods:**

The miR-21 expression levels were quantified (qRT-PCR) in a panel of 86 cases of invasive breast carcinomas in relation to metastasis free survival. The cellular radiosensitivity of human breast cancer cells after irradiation was determined comparing two cell lines (T47D and MDA-MB-361) by cell proliferation and colony forming assays. The influence of miR-21 overexpression or downregulation on cell cycle progression and G2/M checkpoint arrest after irradiation was assessed by flow cytometric analysis.

**Results:**

The expression of miR-21 was transiently increased 8 hours after irradiation in the radioresistant T47D cells and significantly changed with lower extent in radiosensitive MDA-MB-361 cells. Anti-miR-21 treated breast cancer cells failed to exhibit the DNA damage-G2 checkpoint increase after irradiation. Apoptotic activity was significantly enhanced from 7% to 27% in T47D cells and from 18% to 30% in MDA-MB-361 cells 24 hours after 5 Gy irradiation. Additionally, we characterized expression of miR-21 in invasive breast carcinomas. In comparison to non-cancerous adjacent breast tissue, tumours samples had increased miR-21 expression that inversely correlated with the distant metastases-free survival of patients (p = 0.029).

**Conclusions:**

Our data indicate that miR-21 expression in breast cancer cells contributes to radiation resistance by compromising cell cycle progression. These data point to the potential of combining radiotherapy with an anti-miR-21 as a potent G2/M check point inhibitor in overcoming radiation resistance of tumours.

## Background

MicroRNAs (miRNAs) are functional small nucleic acids that regulate the stability and translational efficiency of target messenger RNAs [1]. Altered expression of mi-RNAs has been demonstrated in several human cancers where miRNA 'signatures' are found to be informative for tumour classification and clinical outcome [2,3]. Although several miRNAs are upregulated in specific tumour types [4], a global reduction of miRNA abundance is the more common trait in human cancers. This results in considerable influence on the transformed phenotype [5]. In human breast cancer the deregulation of miRNA expression was first demonstrated by Iorio et al. [6], who suggested a possible role for miRNAs as robust biomarkers for breast cancer diagnosis and prognosis. Recent studies [7] provide evidence that miRNAs are involved in many of the cellular regulatory processes, including activation of different signaling pathways and induction of apoptosis [8,9].

The heterogeneity of human cancers requires the use of multiple therapeutic modalities, including radiation therapy. However, the development of radioresistance presents a problem over prolonged courses of treatment [7,10]. Only a few studies have described the effect of radiation on miRNA expression profiles [7,11,12]. There are indications that radiation sensitivity may be manipulated by influencing the expression of a single miRNA species [11,13,14]. However, little is known of the underlying mechanisms. A more detailed knowledge about radiation influences on miRNA expression in tumour cells is important for improving the effectiveness and reducing the side effects of radiotherapy. Overexpression of miR-21 has been previously reported [15-17]. Patients with high tumour miR-21 expression have a worse clinical outcome than those with low tumour miR-21 expression [18]. A possible explanation was provided by a genome wide search for miR-21 targets. This suggested a functional link between miR-21 and the p53 tumour suppressor pathway [17,19], where p53-induced proteins provoke apoptosis in response to DNA damage after irradiation in cancer. One possibility to improve therapeutic strategy is the modulation of cell cycle progression. The fact that the radiation-induced G2-phase block is a universal event in tumour cells renders the G2/M checkpoint as target for improved efficacy of radiation therapy [20]. Most of the cancer cells have mutations in genes involved in the G1 checkpoint such as p53, Rb, p16, MDM2 and cyclin D1 [21,22]. Interestingly the G2 checkpoint is usually retained in the cancer cells with impaired G1 checkpoint. Therefore if the G2 checkpoint is selectively disrupted the cancer cells with impaired G1 checkpoint would become more sensitive to the DNA-damaging treatment compared with normal cells because normal cells still retain G1 checkpoint intact [21].

In this study, we characterized expression of miR-21 in 86 invasive mammary carcinomas, supporting poor prognostic effects with high miR-21 expression. Additionally, we identify changes in miR-21 levels and cellular response regulation after irradiation in breast cancer cells. Furthermore, we give evidence that modulating the miR-21 expression level would be an important milestone in efficient breast cancer radiation therapy treatment.

## Methods

### Growth and maintenance of cell lines

The breast cancer cell line MDA–MB–361 was cultured in DMEM (Dulbecco Modified Eagles medium) with 20% FCS, (Invitrogen, Carlsbad, CA) and T47D was maintained in RPMI 1640 (Roswell Park Memorial Institute medium) supplemented with 10% FCS and human insulin (10 μg/ml). The cell cultures were maintained in a water humidified 37°C incubator with 5% CO_2_.

### Ionizing radiation treatment

Irradiation of cell cultures containing 1 × 10^6^ log phase cells was performed with a Cs-137 irradiator (HWM D-2000, Siemens, Germany) at a dose rate of 0.95 Gy/min. Doses of 2.5 Gy; 5.0 Gy or 7.5 Gy were administered at room temperature and control cells were sham irradiated. The exposed and sham irradiated cells were subsequently incubated at 37°C and harvested after indicated time points for RNA and protein isolation. The experiment was repeated for each dose in triplicate.

### Lentivirus production and infection of breast cancer cell lines

Replication-defective lentiviral particles were produced by transient co-transfection of HEK293T cells in a 10 cm petri dish with 16 μg, 8 μg and 4 μg of packaging plasmids pMDLg/pRRE, pRSV. Rev and pMD2.G (a kind gift from D. Trono, École polytechnique fédérale de Lausanne) and 8 μg of lentiviral transduction vector pGreenPuro (pGP; System Biosciences, California) using Lipofectamine 2000 (Life Technologies, California) according to the manufacturer’s instructions. The pGP vector (named EV – empty virus in results section) was used as the backbone for miR-21 overexpression and miR-21 downregulation (anti-miR-21) by specific miRNA oligo cloning (pmiRZIP-21 - Cat. Nr. MZIP21-PA-1-GVO-SB; Biocat, Heidelberg, Germany).

The virus particles were harvested 48 hours after transfection, cleared and concentrated as previously described [23]. According to virus titer determination virus productions ranged between 10^8^ and 10^9^ TU/ml (TU - Transduction Units). Viral infection of breast cancer cells was performed using protocols previously described [24]. Briefly, 2 × 10^5^ cells per well were infected with 4 × 10^5^ TU/ml (defined as 2 MOI – multiplicity of infection) and three days after infection GFP expression was monitored. After infection 5 × 10^5^ cells were irradiated for indicated time points. Microscopic analysis was done 48 hours post irradiation (HBO 50/AC and AxioCam MRC, Carl Zeiss AG, Germany).

### RNA isolation for miRNA expression analysis

Paraffin-embedded tissue was microdissected with a sterile needle from 5 μm thick sections using a stereo microscope (Stemi 2000, Zeiss, Germany). A consecutive H&E-stained section was used for guidance. Tumour cell material (containing at least *>*80% tumour cells) was collected from all cases. Additionally, histologically normal ductal epithelium material was collected from five cases as control tissue. Total RNA was isolated from microdissected tissues as previously described [25]. After digestion in lysis buffer and 500 μg/ml proteinase K the RNA was purified by phenol/chloroform extraction, ethanol precipitated, and dissolved in 20 μl RNase-free water. Five microlitres (100 ng) of RNA were reverse-transcribed using MultiScribeTM reverse transcriptase (Applied Biosystems; Foster City, CA, USA) [26]. Further processing and evaluation of the results was performed according to the manufacturer’s instructions.

Total RNA was isolated from each of the breast cancer cell lines (MDA-MB-361 and T47D) after irradiation. Cells were pelleted by centrifugation at 1500 rpm for 5 min, and washed with 1 ml Dulbecco’s phosphate-buffered saline (PBS) without MgCl_2_ and CaCl_2_ (Invitrogen, Carlsbad, CA, USA). Small RNAs (<200 nucleotides) were isolated from the cells using the mirVana™ miRNA isolation kit (Applied Biosystems; Foster City, CA, USA) following the protocol for total RNA isolation. The quantity and quality of the total RNA and miRNA was measured with the Nanodrop spectrophotometer (PeqLab Biotechnology; Germany) and by running 2% agarose gels stained with ethidium bromide, respectively.

### TaqMan-miRNA assays and data analysis

A specific single TaqMan – miRNA assay (Applied Biosystems, Forster City, CA, USA) was used for miR-21 expression analysis (Cat.Nr. 4427975; Assay ID 000397) in total RNA isolations from FFPE samples and from cells treated with irradiation. Quantitative PCR was performed on StepOnePlus Detection System (Applied Biosystems, Foster City, CA) according to the manufacturer’s instructions. The relative expression values of specific miRNA were calculated by using the 2^–ΔΔCT^ method [27] normalized to the control miRNA (RNU43 and RNU44 - Cat.Nr. 4427975; Assay ID 001094 and 001095) and to the FFPE control or non-irradiated sample. All reactions were performed at least twice in duplicate.

### Cell Proliferation and survival

Cell proliferation and viability was determined with a colorimetric cell proliferation WST1 kit (Roche, Manheim, Germany). Twenty-four hours before irradiation, 1000 to 2000 cells per well were seeded into 24-well plates. Three days after irradiation, 200 μl fresh growth medium and 20 μl WST1 labeling reagent were added and the cells were incubated for 2 hours in a 37°C incubator with 5% CO_2_. After incubation the absorbance was determined at 450 nm with reference length at 650 nm using a spectrophotometer plate reader (TECAN, Switzerland). For the measurement of clonogenic survival, cells were seeded in range of densities (500–2000 cells per plate) and 24 h later irradiation was performed. After 10–14 days, the colony formation capacity was assayed after ethanol fixation and Giemsa staining.

### Cell cycle and subG1 fraction analysis

DNA staining of isolated nuclei for cell cycle analysis was performed using a modification of the method of Nüsse et al., [28]. At each indicated time, the treated cells were trypsinized and collected by centrifugation at 300 g for 5 min, and the supernatant was carefully removed. The cell pellet was gently resuspended in 500 μl of a solution containing 10 mM NaCl, 4 mM Na-citrate, 10 μg/ml RNase, 0.3% Nonidet P-40, and 50 μg/ml propidiumiodide (PI). The cell suspensions were incubated for 60 min at room temperature followed by the addition of 500 μl of solution containing 70 mM citric acid, 250 mM sucrose and 50 μg/ml PI. The cell suspensions were mixed and stored at 4°C before flow cytometry. Cell cycle distributions were analyzed on a FACScan LSR II (Becton- Dickinson) (excitation wavelength: 488 nm; emission wavelength: 610 nm, LSR II, Becton Dickinson/FACS DIVA Software). Cells with a DNA content less than that of cells in the G1 phase of the cell cycle (<2n) were assigned to the subG1 fraction and were considered to be apoptotic.

### Patients and tumour samples

Formalin-fixed and paraffin-embedded (FFPE) archival material, obtained from 86 patients with invasive ductal breast carcinomas (IDC), was used for miRNA analysis. Forty-nine tumours were lymph node negative and 57 tumours were small in size (≤2 cm). Nine of the tumours were histological grade 1, 56 were grade 2, and 23 were grade 3 [29,30]. The patients age ranged from 15 to 84 years (median 66 years). All patients were surgically treated, and no patient received preoperative adjuvant chemotherapy treatment. Postoperative 29 patients received radiation therapy treatment and 4 patients received Novaldex with radiation therapy. Detailed long-term clinical follow-up was available for all patients with a median follow-up period of 113 months (min. 5 months, max. 468 months). Forty patients relapsed with distant metastases within the total follow-up period. Ethical approval for the study was obtained from the Ethics Committee of the Medical Faculty of the Technical University of Munich.

### Statistics

Correlation between histopathological markers and miRNA expression was examined by Spearman's rank correlation test. For univariate survival analysis Kaplan-Meier curves were calculated from 86 patients, and differences between strata were tested with the log-rank Chi-Square value. Results obtained in the *in vitro* experiments were tested using one- or two-way ANOVA and GraphPad Prism. In all analysis statistical significance was considered at the p <0.05 levels.

## Results

### Breast cancer cellular characterisation after irradiation

**Figure 1:**
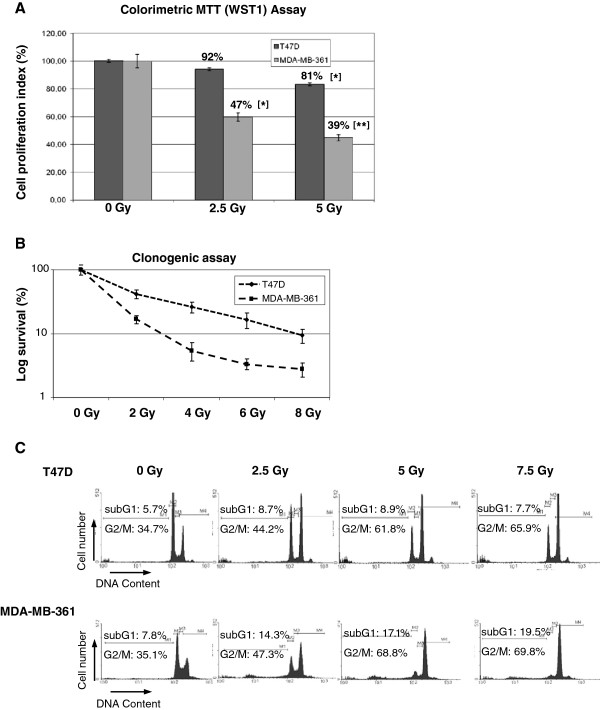
**Breast cancer cell survival and cell cycle characterisation after irradiation. (A)** Growth characteristics of T47D and MDA-MB-361 breast cancer cells were determined by MTT (WST1) assay 72 hours after irradiation. Data represent the means ± SD (n=4). *p <0.05, **p <0.01 by ANOVA one-way analysis of variance. **(B)** clonogenic survival of breast cancer cells 10 days after irradiation. **(C)** Cell cycle progression in breast cancer cells was evaluated by PI staining and flow cytometry 24 hours after irradiation at the indicated doses.

Two breast cancer cell lines (T47D and MDA-MB-361) were analysed for their radiation sensitivity. Seventy-two hours after 2.5 Gy and 5 Gy irradiation the cellular proliferation activity was determined by MTT (WST1) assay (Figure 1A). After 5 Gy irradiation the MDA-MB-361 cells showed greatly reduced survival (39%) in comparison to T47D cells (81% survival) and mock irradiated control (settled as 100%). To confirm the increased irradiation sensitivity of MDA-MB-361 cells we measured clonogenic survival (Figure 1B). Here we observed the expected reduced survival capacity of MDA-MB-361 cells (colony formation) 10 days after irradiation (Figure 1B). Cell cycle distribution was monitored by FACS analysis of DNA content 24 hours after irradiation (Figure 1C). With increasing radiation doses both cell lines displayed an accumulation of cells arresting at G2/M, accompanied by a reduction of cells in G1. The extent of the G2/M accumulation was greater in the radiation sensitive MDA-MB-361 cells, with almost 69% of cells in G2/M phase after 5 Gy irradiation. In irradiated T47D cells 62% of cells were in G2/M phase at the same time point.

**Figure 2:**
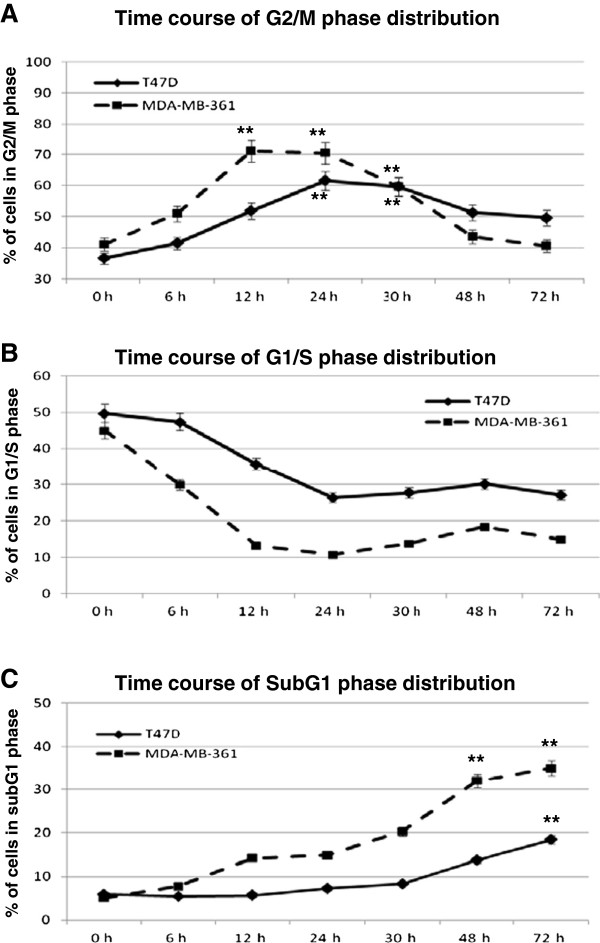
**Cell cycle time kinetics in breast cancer cells after 5 Gy irradiation.** Cell cycle distributions were analyzed by FACS and changes in cellular fractions of G2/M **(A)**, G1/S **(B)** and subG1 **(C)** after indicated time points are presented. Data represent the means ± SD (n=2). *p <0.05, **p <0.01 by ANOVA one-way analysis of variance.

The time course of the G2/M phase accumulation was monitored after 5 Gy irradiation (Figure 2A), showing faster and more prominent G2/M accumulation for MDA-MB-361 cells with a peak after 12 hours. These changes were accompanied with faster reduction in G1 phase (Figure 2B) and the appearance of a subG1 fraction of apoptotic cells already 12 hours after irradiation. In T47D cells the changes were less prominent, but slight increase in subG1 fraction was nevertheless detectable 72 hours after irradiation (Figure 2C). These results establish the T47D cells as radioresistant and MDA-MB-361 cells as radiosensitive cell line.

### Characterization of miR-21 expression after irradiation

**Figure 3:**
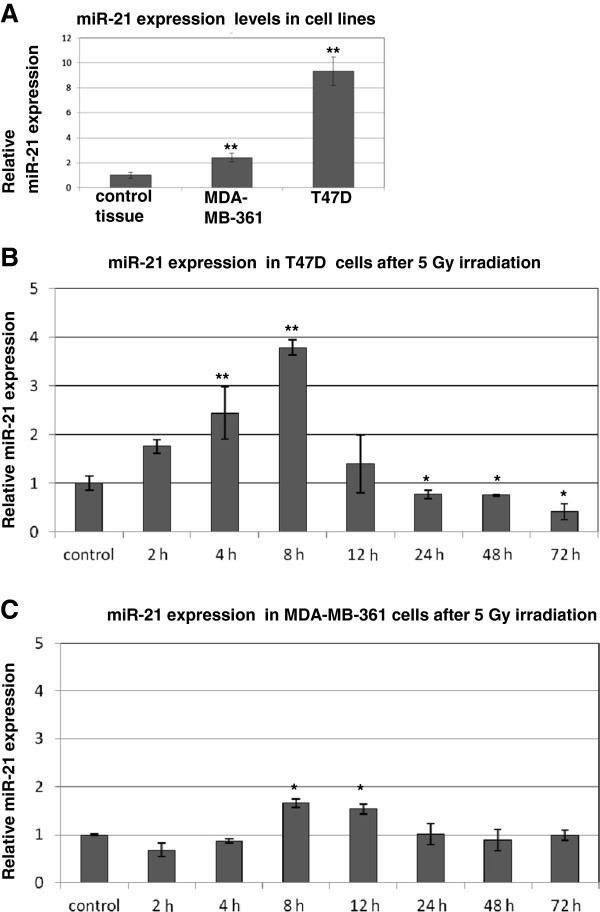
**Time kinetics of miR-21 expression in breast cancer cells after 5 Gy irradiation. (A)** Relative miR-21 expression in breast cancer cell lines compared to adjacent control mammary tissue. **(B)** Relative miR-21 expression in T47D and **(C)** MDA-MB-361 breast cancer cells after 5Gy irradiation and indicated time points in relation to the expression of sham irradiated controls. Data represent the means ± SD (n=4). *p <0.05, **p <0.01 by ANOVA.

**Figure 4:**
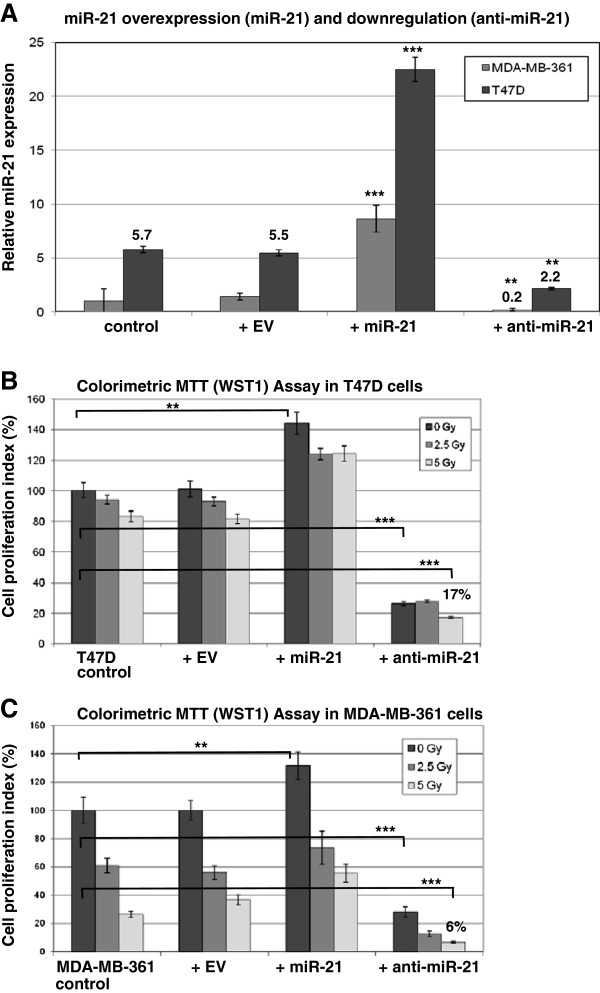
**miR-21 downregulation regulates the cellular response to radiation. (A)** T47D cells were infected with empty lentivirus (+ EV), with miR-21 overexpressing LV (+ miR-21) or inhibitory miR-21 LV (+ anti-mir-21) and analysed for miRNA expression in MDA-MB-361 cells (light gray boxes) and T47D cells (dark gray boxes) 72 hours after infection. MDA-MB-361 control sample was used as reference for miR-21 expression (set as 1). **(B)** Cell proliferation assay (WST1) in T47D cells with miR-21 overexpression or downregulation and corresponding radiation treatment after 72 hours in control cells (dark gray boxes), or after 2.5 Gy (gray boxes) and 5 Gy (light gray boxes) 72 hours after irradiation. **(C)** Cell proliferation assay (WST1) in MDA-MB-361 cells with miR-21 overexpression or downregulation and corresponding radiation treatment after 72 hours. Data represent the means ± SD (n=3). *p <0.05, **p <0.01, ***p <0.001 by ANOVA.

Increased miR-21 expression levels were detected in both cell lines (T47D and MDA-MB-361) compared to control adjacent mammary tissue (Figure 3A). Interestingly the resistant T47D showed fivefold higher miR-21 expression than MDA-MB-361 (Figure 4A).

In order to determine if miR-21 expression is influenced by ionizing radiation, the miR-21 levels were measured in exponentially growing breast cancer cells (T47D and MDA-MB-361) following 5 Gy irradiation (Figure 3B and C). MiR-21 expression showed a prominent induction 8 hours after irradiation only in the radioresistant T47D cells. These transient changes in miR-21 expression levels correlate with postponed lower apoptotic subG1 fraction accumulation in the T47D cells 72 hours after irradiation (18% compared to 35% in the MDA-MB-361 cells on Figure 2C). This suggests that miR-21 transient increase inhibits apoptotic cellular response in radiation resistant T47D cells.

### Functional analysis of miR-21 overexpression and inhibition in breast cells

To repress miR-21 expression we infected T47D and MDA-MB-361 cells with a lentiviral anti-miR-21 vector (anti-miR-21). An empty lentivirus (EV) was used as a control in parallel with a lentivirus overexpressing miR-21. The level of mature miR-21 in infected and control cells was measured by quantitative RT-PCR and correlated to MDA-MB-361 as 1 fold expression control (Figure 4A). In T47D cells miR-21 expression was 5.5 fold higher than in MDA-MB-361 cells. The miR-21 overexpression produced 4 fold higher levels of miR-21 when compared to untreated T47D cells and cells infected with control virus (EV) 72 hours post infection (dark gray boxes, Figure 4A). Consequently, miR-21 expression was decreased by anti-miR-21 lentiviral infection as compared to control cells (T47D and EV). Nevertheless the inhibition of miR-21 expression in T47D cells was 2.2 fold higher than miR-21 expression levels in untreated MDA-MB-361 cells. Accordingly, miR-21 overexpression and downregulation data were analyzed in MDA-MB-361 cells (light gray boxes, Figure 4A) with more prominent inhibition of miR-21 expression levels using anti-miR-21 and compared to MDA-MB-361 control cells.

Cell proliferation was measured by MTT (WST1) assay 72 hours after seeding exeperiments in T47D (Figure 4B) and MDA-MB-361 cells (Figure 4C). MiR-21 overexpression slightly increased cell proliferation (140%) and miR-21 inhibition dramatically reduced cellular proliferation to 31% (dark gray boxes, Figure 4C and D), in both breast cancer cell lines analyzed.

### Functional analysis of miR-21 overexpression and inhibition in breast cancer cellular response to radiation

Since miR-21 is well expressed in non irradiated T47D cells (Figure 3A) and is dramatically elevated by radiation we proposed that inhibition of miR-21 would decrease the degree of radiation resistance. Consequently, 24 hours after irradiation with 2.5 and 5 Gy the relative miR-21 expression levels showed decrease in expression (~ 40%) in control cells and cells overexpressing miR-21 (Additional file 1: Figure S1 **(A)**. In the cells with miR-21 inhibition by anti-miR-21 no detectable change to the suppressed miR-21 level was detectable after irradiation at RNA detection levels (light gray boxes, Additional file 1: Figure S1**(A)** and **(B)**. Representative micrographs of T47D and MDA-MB-361 cells 72 hours after 5 Gy irradiation in the presence of miR-21 overexpression or inhibition are shown in Additional file 1: Figure S1 **(C)** and **(D)**. Knockdown of miR-21 leads to a decrease in adherent cells and a concomitant increase in detached cells (presumed to be non-viable cells).

Cell proliferation was measured by MTT (WST1) assay 72 hours after 2.5 Gy and 5 Gy irradiation in T47D (Figure 4B) and MDA-MB-361 cells (Figure 4C). The changes after irradiation in miR-21 overexpression T47D cells were comparable to the changes in control cells or cells infected with control virus (EV), showing slightly increased proliferation rate after miR-21 overexpression and irradiation. Downregulation of miR-21 expression dramatically reduces cellular proliferation and this effect was more pronounced after irradiation (Figure 4B and C, light gray boxes). Anti-miR-21 influenced cellular proliferation with additional decrease in proliferation after 5Gy irradiation in MDA-MB-361 cells (6%). With anti-miR-21 treatment and 5Gy irradiation proliferation rate in T47D cells was 17% what correlates with 2.2 fold miR-21 expression levels in T47D cells compared to MDA-MB-361 cells after miR-21 knockdown (Figure 4A).

### MiR-21 knockdown results in loss of radiation-induced G2/M arrest

**Figure 5:**
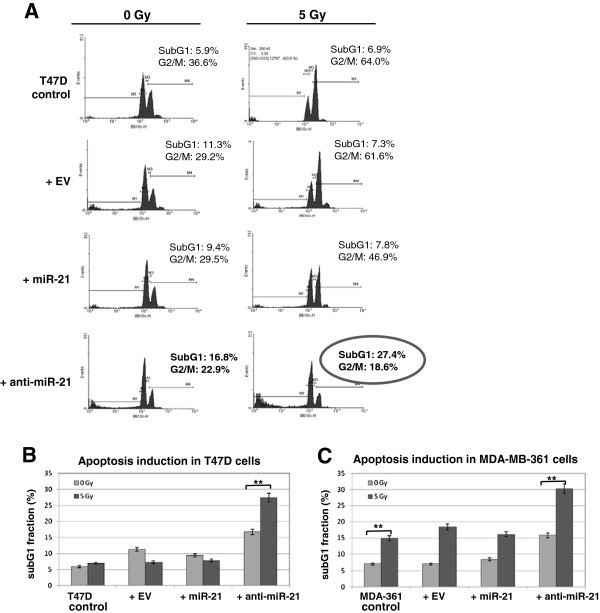
**miR-21 downregulation abrogates G2/M check point accumulation after irradiation. (A)** T47D cells were infected with empty lentivirus (+ EV), with miR-21 overexpressing LV (+ miR-21) or inhibitory miR-21 LV (+ anti-mir-21) and analysed for cell cycle changes 24 hours after 5 Gy irradiation. One representative FACS analysis is shown from three independent experiments. **(B)** Statistical analysis of subG1 cellular fraction in T47D infected cells (as in part A) and **(C)** in MDA-MB-361 cells (control cells - light gray boxes) or after 5 Gy irradiation (dark gray boxes). Data represent the means ± SD (n=3). *p <0.05, **p <0.01 by ANOVA.

Previously, it has been shown that miR-21 affects cell cycle progression, cellular proliferation and migration in human breast cancer cells [31]. In agreement with these results we observed that miR-21 knockdown reduces the number of cells in the G2 phase from 36.6% in control cells to 22.9% in anti-miR-21 T47D lentivirus infected cells (Figure 5A). This modest reduction became more prominent after irradiation in both cell lines analyzed (Figure 5A and Additional file 2: Figure S2). In T47D cells the G2/M fraction 24 hours after irradiation fell from 64% in control cells to 18.6% (Figure 5A). Interestingly, the G2/M radiation checkpoint abrogation due to miR-21 knockdown (anti-miR-21) is persistent (6.9%) 72 hours after irradiation (Additional file 3: Figure S3 **(A)**. A significant increase in subG1 cellular apoptotic fraction from 6.9% (8.9% in Figure 1C) to 27.4% (24 hours after) and to 49.3% (72 hours) after 5Gy irradiation is detected (Figure 5A and Additional file 3: Figure 3A). Increase in subG1 fraction was significantly enhanced in T47D cells and comparable to radiosensitive cellular response of MDA-MB-361 cells (Figure 5C). These data confirm that both radiation sensitive and radiation resistant cancer cell line show a prominent increase in subG1 cellular fraction after miR-21 knockdown combined with irradiation and further point importance of synergistic effects of miR-21 inhibition with radiotherapy.

### MiR-21 expression and prognosis in breast cancer

**Figure 6:**
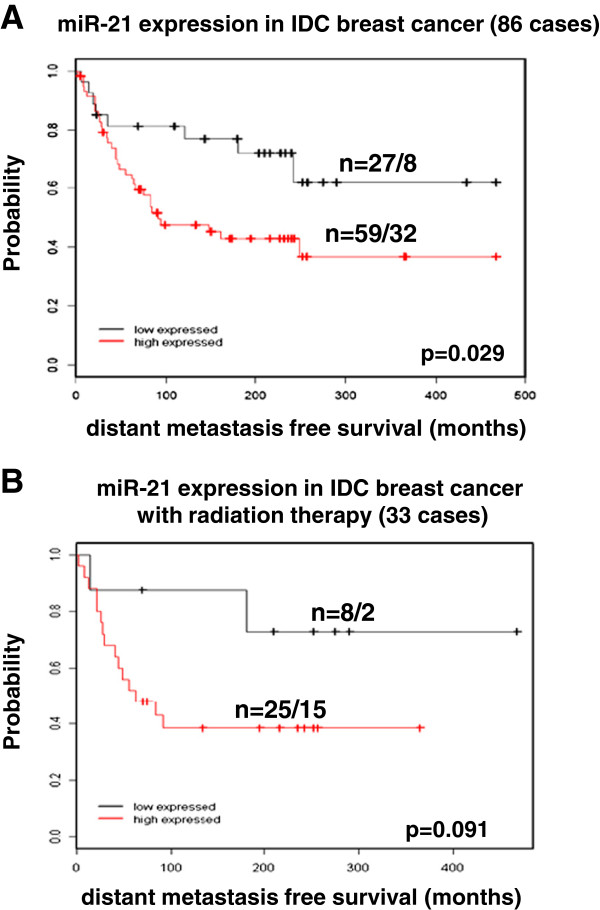
**miR-21 expression in breast cancer. (A)** Kaplan Meier analyses for distant metastasis-free survival of patients. The patients were grouped into those with low (<1.8-fold) and high (>1.8-fold) miR-21 expression in tumour tissues, with a significantly better prognosis for patients with low miR-21 expression (p = 0.029). **(B)** Expression of miR-21 in radiotherapy treated patients group. Although this analysis did not reach significance (p = 0.091) a clear trend is obvious for better prognosis of patients with low miR-21 expression.

The expression of miR-21 in breast tumours was determined in 86 cases of invasive breast carcinomas with long-term follow-up [30]. In comparison to normal adjacent tissue samples, the breast cancers showed increased expression of miR-21 (median 1.4 fold). Higher levels of miR-21 expression significantly correlated with lower distant metastases-free survival of patients (p = 0.029) (Figure 6A). From 86 cases 33 received postoperative radiation treatment, whereas 25 from 59 patients with high miR-21 expression and eight from 27 patients with low miR-21 expression were treated with radiation therapy (Figure 6B). A trend towards better prognosis and increased survival in patients with low miR-21 expression receiving radiation therapy was evident, but will require validation with a much larger patient collective.

## Discussion

Upregulation of miR-21 is a frequent miRNA alteration described in human cancers [32]. The consequences of overexpression of miR-21 is that it acts as an “oncomir” blocking apoptosis [33], promoting cell proliferation [18,34] and causing invasion and metastasis [35,36]. It appears that miR-21 targets multiple tumour-supressive pathways [31] and recent studies showed convincing evidence that miR-21 negatively regulates Cdc25A and cell cycle progression in colon cancer [37] and in human glioblastoma cells [38]. According to miR-21 target analysis, Lu et al., demonstrated that miR-21 promotes cell transformation by targeting the programmed cell death 4 gene (PDCD4) in MCF7 breast cancer cells [39]. There is some evidence that miRNAs are also involved in modulating radiation sensitivity in lymphoblastic cell lines [7], endothelial cells [14] and for resistance to cytotoxic anticancer therapy in lung cancer cells [11]. Data published recently from Gwak et al., [40] demonstrate the importance of miR-21 knockdown in radiosensitation of glioblastomas. In correlation with our results they present importance of high miR-21 expression levels in conferring radiation resistance in glioblastomas. The roles of miR-21 expression in modulating response of breast tumour cells to irradiation have not been previously analyzed.

Therefore, we have investigated miR-21 expression in radiation resistant and radiation sensitive breast cancer cells after exposure to γ-irradiation. We observed that the expression of miR-21 was not significantly changed after 5 Gy exposure of the radiosensitive MDA-MB-361 cells, but was transiently increased in radiation resistant T47D cells. This data support hypothesis that miR-21 is not merely upregulated in association with oncogenesis, but rather can act as radioresistant miRNA when transiently overexpressed after radiation treatment [36].

The G2/M checkpoint arrest is prominent after exposure to DNA damage reagents such as γ-irradiation [21,41]. Our cell cycle data analysis showed that the anti-apoptotic action of miR-21 is also evident after radiation exposure and correlates with radiation resistance. In addition, miR-21 influence cell cycle progression via the DNA damage-G2 checkpoint induction. In this matter miR-21 inhibition (anti-miR-21) is able to reduce the G2/M block and to enhance apoptosis induction 24 hours after radiation treatment (Figure 5A). All together these data suggest the importance of combination therapy such as radiotherapy with efficient G2/M check point inhibitor anti-miR-21. Supporting our results in the manuscript of Li et al. [38], it is presented that miR-21 inhibitor reduces G2/M arrest what is inconsistent with recently published data from Gwak et al., [40] showing G2/M induction after miR-21 knockdown in glioblastoma cells. This highlights the importance of G2/M arrest after radiation treatment to be studied in different tumour cell types to further support a general conclusion about miR-21 function in radioresitance.

The data presented in Figure 6 confirm previously published data from Yan et al. [18], demonstrating increased expression of miR-21 in breast cancer. We identify that patients with low expression levels of miR-21 have better clinical outcome. Previously it has been reported that high levels of miR-21 expression correlate with advanced clinical stage, lymph node metastasis and shortened survival of the patients [18,42]. This is confirmed by the association we observe between low miR-21 expression and distant metastasis free survival. The role of miR-21 in shaping the response to radiotherapy is suggested by the increased clinical survival seen for low miR-21 patient group after radiation therapy.

## Conclusions

Taken together, our results show that miR-21 expression transiently increases in response to irradiation treatment in the T47D radiation resistant cell line. Furthermore, the miR-21 knockdown improved radiation induced apoptosis and growth arrest in radiation resistant cells almost to the same extent as in sensitive breast cancer cells (MDA-MB-361). These findings are important concerning the better clinical outcome for patients with low miR-21 expression levels and the use of miR-21 as potential target in breast cancer therapy.

## Competing interests

The authors declare no potential conflict of interest.

## Authors’ contributions

NA, MA and MJA coordinated the study and drafted the manuscript. NA, IH, IGV, KR, and NL performed experiments and analyzed data. GA contributed important material for analysis. HB performed statistical analysis. All authors read and approved the final manuscript.

## Supplementary Material

Additional file 1: Figure S1 qRT-PCR quantification of miR-21 overexpression and downregulation 24 hours after irradiation. **(A)** T47D and **(B)** MDA-MB-361 cells were infected with empty lentivirus (+ EV), with miR-21 overexpressing LV (+ miR-21) or inhibitory miR-21 LV (+ anti-mir-21) and analysed for miRNA expression changes in control cells (dark gray boxes), or after 2.5 Gy (gray boxes) and 5 Gy (light gray boxes) 24 hours after irradiation. Data represent the means ± SD (n=3). *p <0.05, **p <0.01 by ANOVA. **(C)** Representative micrographs (scale bar = 50 μm) of T47D cells and **(D)** MDA-MB-361 cells 72 hours after 5 Gy irradiation with miR-21 overexpression (+ miR-21) or inhibition (+ anti-miR-21). Click here for file

Additional file 2: Figure S2 miR-21 downregulation induces considerable cellular apoptosis 24 hours after irradiation in MDA-MB-361 cells. MDA-MB-361 cells were infected with empty lentivirus (+ EV), with miR-21 overexpressing LV (+ miR-21) or inhibitory miR-21 LV (+ anti-miR-21) and analysed for cell cycle changes 24 hours after 5 Gy irradiation. One representative FACS analysis is shown from three independent experiments. Click here for file

Additional file 3: Figure S3 miR-21 downregulation induces considerable cellular apoptosis 72 hours after irradiation in T47D cells. **(A)** T47D cells were infected with empty lentivirus (+ EV), with miR-21 overexpressing LV (+ miR-21) or inhibitory miR-21 LV (+ anti-mir-21) and analysed for cell cycle changes 72 hours after 5 Gy irradiation. One representative FACS analysis is shown. **(B)** Statistical analysis of subG1 cellular fraction in T47D infected cells (control cells - light gray boxes) or after 5 Gy irradiation (dark gray boxes). Data represent the means ± SD (n=3). *p <0.05 by ANOVA. Click here for file
