# Supplementary figures and images for: Radiation resistance due to high expression of miR-21 and G2/M checkpoint arrest in breast cancer cells
Source: Radiat Oncol. 2012 Dec 5;7:206. doi: 10.1186/1748-717X-7-206 (PMC3573984; doi:10.1186/1748-717X-7-206)

A

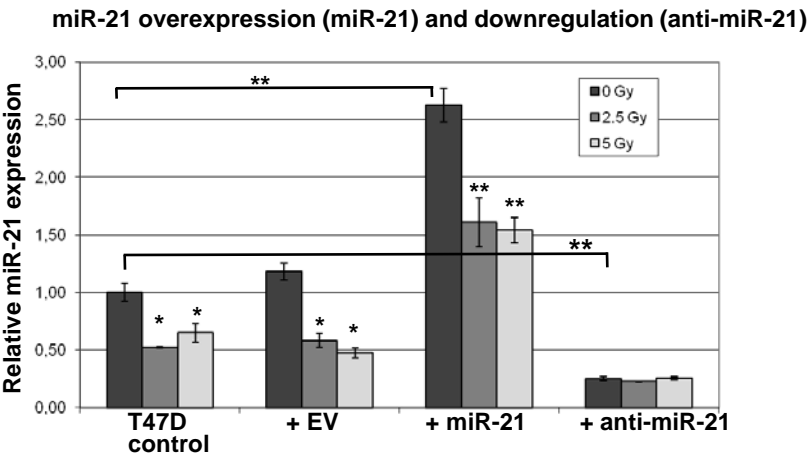

B

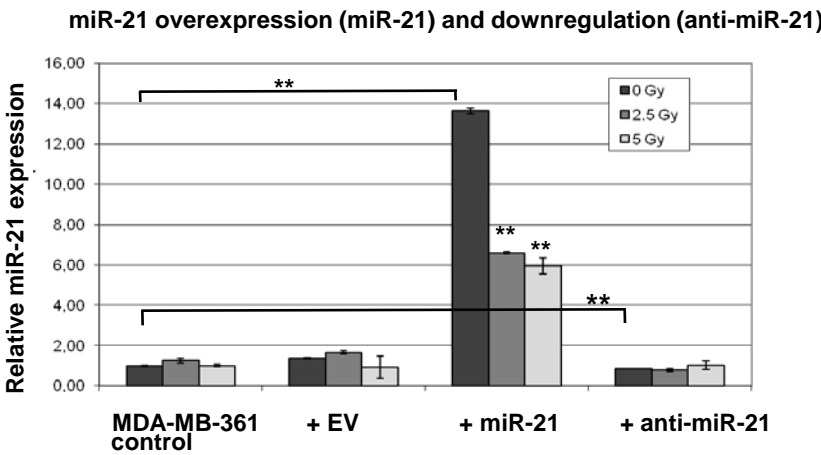

C

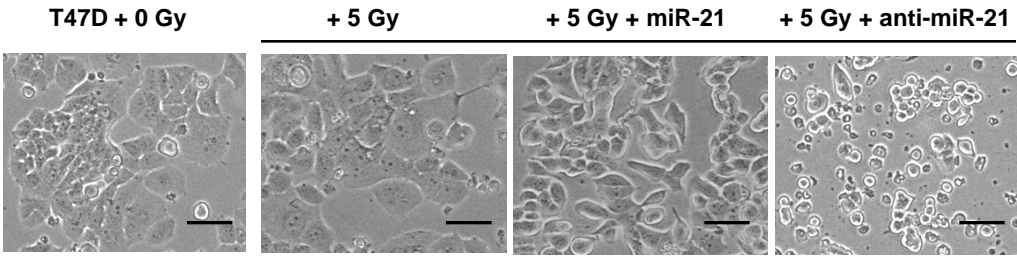

D

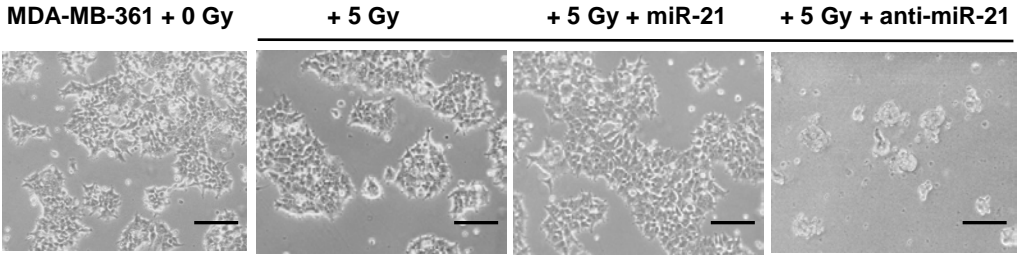

Supplement: Additional file 1: Figure S1 — qRT-PCR quantification of miR-21 overexpression and downregulation 24 hours after irradiation. (A) T47D and (B) MDA-MB-361 cells were infected with empty lentivirus (+ EV), with miR-21 overexpressing LV (+ miR-21) or inhibitory miR-21 LV (+ anti-mir-21) and analysed for miRNA expression changes in control cells (dark gray boxes), or after 2.5 Gy (gray boxes) and 5 Gy (light gray boxes) 24 hours after irradiation. Data represent the means ± SD (n=3). *p <0.05, **p <0.01 by ANOVA. (C) Representative micrographs (scale bar = 50 μm) of T47D cells and (D) MDA-MB-361 cells 72 hours after 5 Gy irradiation with miR-21 overexpression (+ miR-21) or inhibition (+ anti-miR-21). [file 1748-717X-7-206-S1.pdf]

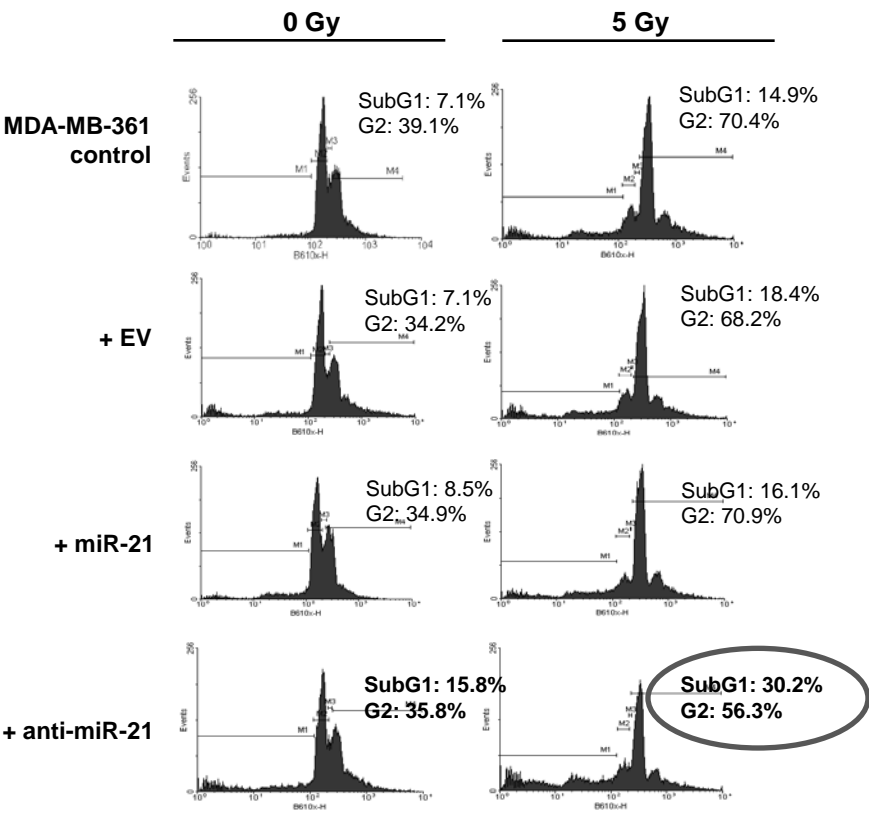

Supplement: Additional file 2: Figure S2 — miR-21 downregulation induces considerable cellular apoptosis 24 hours after irradiation in MDA-MB-361 cells. MDA-MB-361 cells were infected with empty lentivirus (+ EV), with miR-21 overexpressing LV (+ miR-21) or inhibitory miR-21 LV (+ anti-miR-21) and analysed for cell cycle changes 24 hours after 5 Gy irradiation. One representative FACS analysis is shown from three independent experiments. [file 1748-717X-7-206-S2.pdf]

A

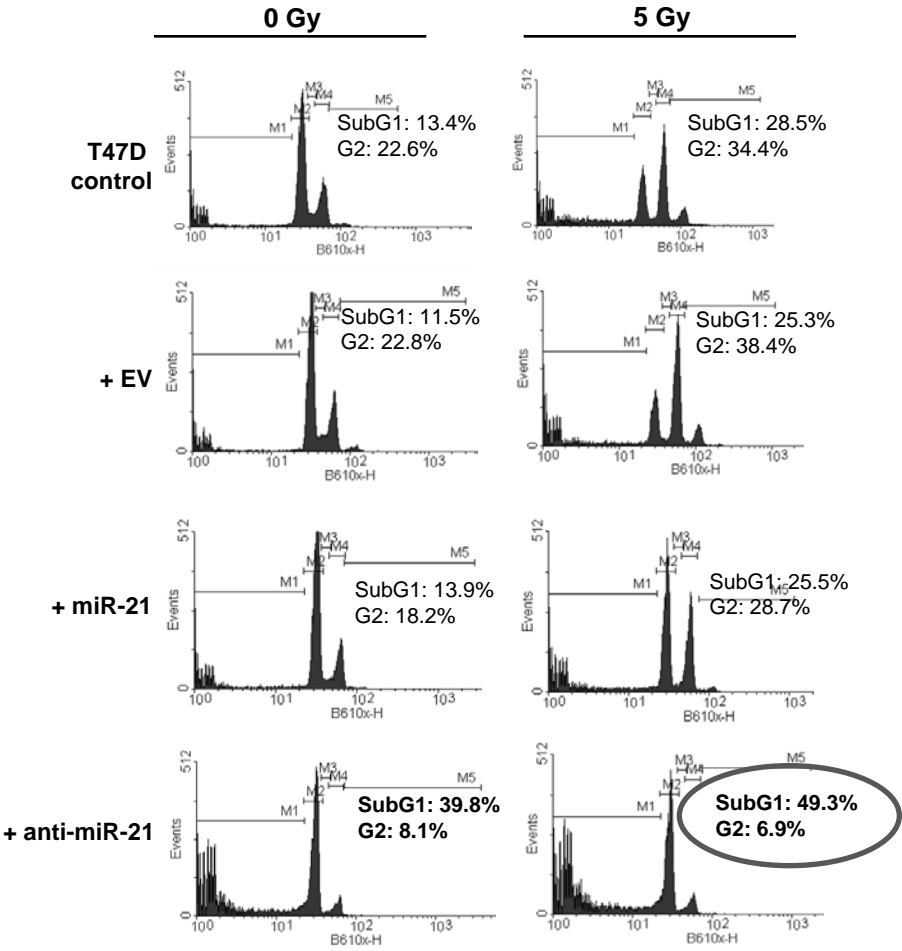

B

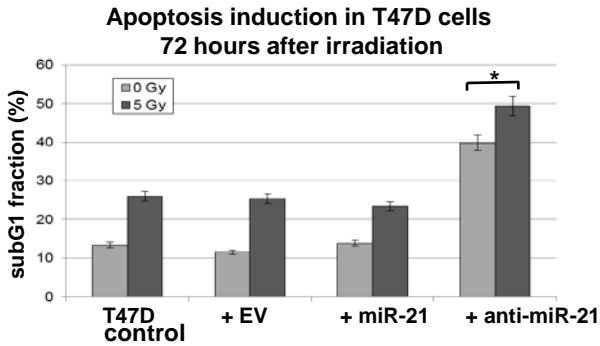

Supplement: Additional file 3: Figure S3 — miR-21 downregulation induces considerable cellular apoptosis 72 hours after irradiation in T47D cells. (A) T47D cells were infected with empty lentivirus (+ EV), with miR-21 overexpressing LV (+ miR-21) or inhibitory miR-21 LV (+ anti-mir-21) and analysed for cell cycle changes 72 hours after 5 Gy irradiation. One representative FACS analysis is shown. (B) Statistical analysis of subG1 cellular fraction in T47D infected cells (control cells - light gray boxes) or after 5 Gy irradiation (dark gray boxes). Data represent the means ± SD (n=3). *p <0.05 by ANOVA. [file 1748-717X-7-206-S3.pdf]
